# Early-Onset Methicillin-Resistant Staphylococcus aureus Blebitis Following PRESERFLO™ MicroShunt Implantation: A Case Report

**DOI:** 10.7759/cureus.93967

**Published:** 2025-10-06

**Authors:** Hiroki Mieno, Morio Ueno, Chie Sotozono

**Affiliations:** 1 Ophthalmology, Kyoto Prefectural University of Medicine, Kyoto, JPN

**Keywords:** blebitis, glaucoma, infection, methicillin-resistant staphylococcus aureus (mrsa), preserflo

## Abstract

We describe a rare case of early-onset bleb-related infection (BRI) caused by methicillin-resistant Staphylococcus aureus (MRSA) following implantation of the PRESERFLO^™^ MicroShunt (PMS; Santen Pharmaceutical, Osaka, Japan). A 70-year-old female with bilateral primary open-angle glaucoma, pseudophakia, and a 15-year history of glaucoma treatment underwent PMS implantation in her left eye. On postoperative day (POD) 25, blebitis was observed, characterized by conjunctival hyperemia, mucopurulent discharge, and eyelid swelling, despite routine postoperative prophylaxis with topical antibiotics. A positive Seidel test result was also observed. Initial management with intensive topical antibiotics was followed by intracameral vancomycin and ceftazidime on POD 27 due to the condition worsening. Cultures of conjunctival discharge grew MRSA, prompting intensive topical and systemic vancomycin therapy, along with levofloxacin. Although ocular inflammation improved, tube exposure occurred on POD 32, necessitating PMS removal and bleb revision. Two months later, the conjunctiva was completely healed, Best-corrected visual acuity (BCVA) was 1.0, and intraocular pressure stabilized at 16 mmHg with a single medication and without any residual sequelae.

Although PMS implantation is generally associated with fewer bleb-related complications than traditional trabeculectomy, early postoperative BRI may occur, particularly in the presence of bleb leakage. This report highlights that resistant organisms, such as MRSA, can cause severe infection even under standard postoperative prophylaxis, and that clinicians should maintain strict follow-up observation for early signs of infection after PMS implantation and consider the possibility of resistant pathogens to ensure timely and effective management.

## Introduction

Bleb-related infection (BRI) is a potentially vision-threatening complication associated with glaucoma filtration surgery, and it can occur from weeks to decades after the procedure [1,2]. The risk of BRI is reportedly strongly associated with bleb location, a thin bleb, use of an antimetabolite, blepharitis, and bleb leakage [1-7]. It is generally recognized that the spectrum of causative microorganisms in BRI varies according to the timing of the onset [2]. Early-onset BRI is thought to result from intraoperative or early postoperative entry of the host’s resident flora, with the most frequently isolated organisms being coagulase-negative staphylococci, particularly Staphylococcus epidermidis, and Staphylococcus aureus [1,2]. In contrast, late-onset BRI is thought to arise from the transconjunctival migration of transiently present bacteria through thin-walled blebs, most commonly Streptococcus species [1-4].

The PRESERFLO™ MicroShunt (PMS; Santen Pharmaceutical, Osaka, Japan) is a device designed to achieve significant intraocular pressure (IOP) reduction with a less invasive approach than traditional surgeries [8]. PMS implantation has been associated with a reduced incidence of bleb-related complications and a decreased need for reinterventions relative to trabeculectomy [9,10]. However, since PMS implantation creates a filtering bleb and requires intraoperative antifibrotic modulation, the possibility of BRI developing remains. Reports of BRI following PMS implantation are rare [11,12], and early-onset cases have not been well documented. We report a rare case of early-onset BRI caused by methicillin-resistant Staphylococcus aureus (MRSA) following PMS implantation.

## Case presentation

The patient was a 70-year-old female with bilateral primary open-angle glaucoma who was pseudophakic in both eyes and had a 15-year history of glaucoma treatment, including a previous trabeculotomy ab interno performed in her right eye. She was in good general health, with no notable medical history, including diabetes, atopic dermatitis, immunosuppressive conditions, or previous systemic infections. Although she had previously used topical brimonidine, the medication was discontinued due to allergic reactions. Despite ongoing treatment with topical tafluprost-timolol fixed combination, dorzolamide, and ripasudil in both eyes, adequate IOP control was not achieved, and she was referred to our department for surgical intervention. At initial presentation, the IOP was 19 mmHg in both eyes. We first performed PMS implantation in her right eye, which was uneventful and had a favorable postoperative course.

Four months later, given the satisfactory outcome in her right eye, PMS implantation was performed in her left eye according to our standardized PMS implantation technique [13]. Briefly, a superior fornix-based conjunctival peritomy was performed. The Tenon’s capsule was then carefully dissected, and mitomycin C (MMC; 0.4 mg/mL) was applied to the exposed sclera with soaked sponges for five minutes. The area was then thoroughly irrigated with saline water. A tract for PMS insertion into the anterior chamber was then created using a dedicated double-step knife, and the fins of the device were firmly seated and secured within the sclera. After confirming aqueous humor drainage from the distal end of the PMS, the posterior portion of the tube was anchored with 10-0 nylon (Mani, Inc., Tochigi, Japan) to prevent displacement or kinking. A 10-0 nylon stent suture was then placed within the PMS lumen to limit aqueous outflow in the early postoperative period. The Tenon’s capsule and the conjunctiva were then closed with 10-0 nylon, and a subconjunctival injection of dexamethasone (1.65 mg) was administered to complete the procedure. Post surgery, the patient was prescribed a four-times daily administration of corticosteroid and antibacterial eye drops.

**Figure 1:**
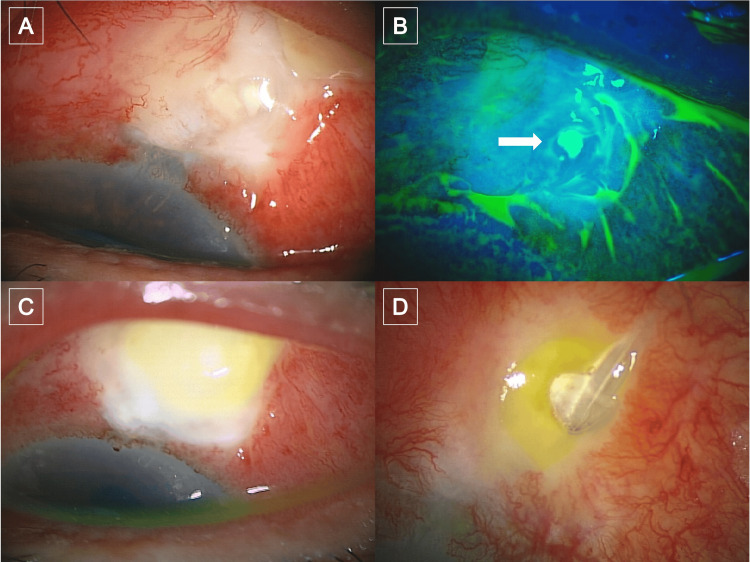
Clinical course following PRESERFLO™ MicroShunt (PMS) implantation in the patient's left eye POD 25: (A) Slit-lamp photograph showing marked localized conjunctival injection surrounding the bleb, with whitish opacification and swelling. (B) Fluorescein-stained slit-lamp photograph demonstrating a positive Seidel-test sign (white arrow). POD 27: (C) Slit-lamp photograph showing expanded purulent infiltration and anterior chamber cells, indicating that the condition was worsening. POD 32: (D) Slit-lamp photograph showing tube exposure, which necessitated PMS removal and bleb revision POD: postoperative day

Following PMS implantation in her left eye, the intraluminal stent was removed on postoperative day (POD) six. On POD 18, the 10-0 conjunctival sutures were removed, at which time Seidel-test positivity was noted, and ofloxacin ointment was added once nightly. As the Seidel positivity was minimal, the anterior chamber depth remained unchanged. On POD 25, she presented with a four-day history of conjunctival hyperemia, a two-day history of mucopurulent discharge, and a one-day history of eyelid swelling. Best-corrected visual acuity (BCVA) was 0.9, and IOP was 15 mmHg. Slit-lamp examination revealed marked localized conjunctival injection surrounding the bleb, accompanied by whitish opacification and swelling of the bleb, with a positive Seidel test at the same site noted on POD 18 (Figures 1A, 1B). However, no anterior chamber inflammation was observed. Topical betamethasone was discontinued, and intensive topical moxifloxacin and cefmenoxime were initiated, with nightly ofloxacin ointment continued. A conjunctival discharge sample was also submitted for culture at that time, and direct smear examination demonstrated Gram-positive cocci. On POD 27, purulent infiltration had expanded (Figure 1C), and a small number of anterior chamber cells were observed, indicating that the condition was worsening.

Consequently, intracameral vancomycin (1 mg/0.1 mL) and ceftazidime (2.25 mg/0.1 mL) were administered. At that time, aqueous humor was also obtained for culture, which was later found to have no growth. On POD 28, there was partial improvement of the symptoms and the eyelid swelling. Cultures from conjunctival discharge grew Staphylococcus aureus, which was confirmed to be MRSA on the same day. On the basis of the culture results, intensive vancomycin therapy was initiated, including topical vancomycin ointment and intravenous administration. Specifically, vancomycin ophthalmic ointment 1% (Toa Pharmaceutical Co., Ltd., Toyama, Japan) was applied six times daily, and intravenous vancomycin (1 g every 12 hours for four doses, followed by one additional dose 24 hours later) was administered, together with levofloxacin eye drops instilled 6 times daily. After those treatments, ocular inflammation showed improvement; however, tube exposure was noted on POD 32 (Figure 1D). Hence, PMS removal, suturing of the scleral tunnel, and conjunctival advancement were performed on the same day. Post surgery, levofloxacin drops, vancomycin ointment, and topical betamethasone were administered and then gradually tapered. Due to an IOP increase after PMS removal, latanoprost-timolol fixed-combination drops were initiated.

**Figure 2:**
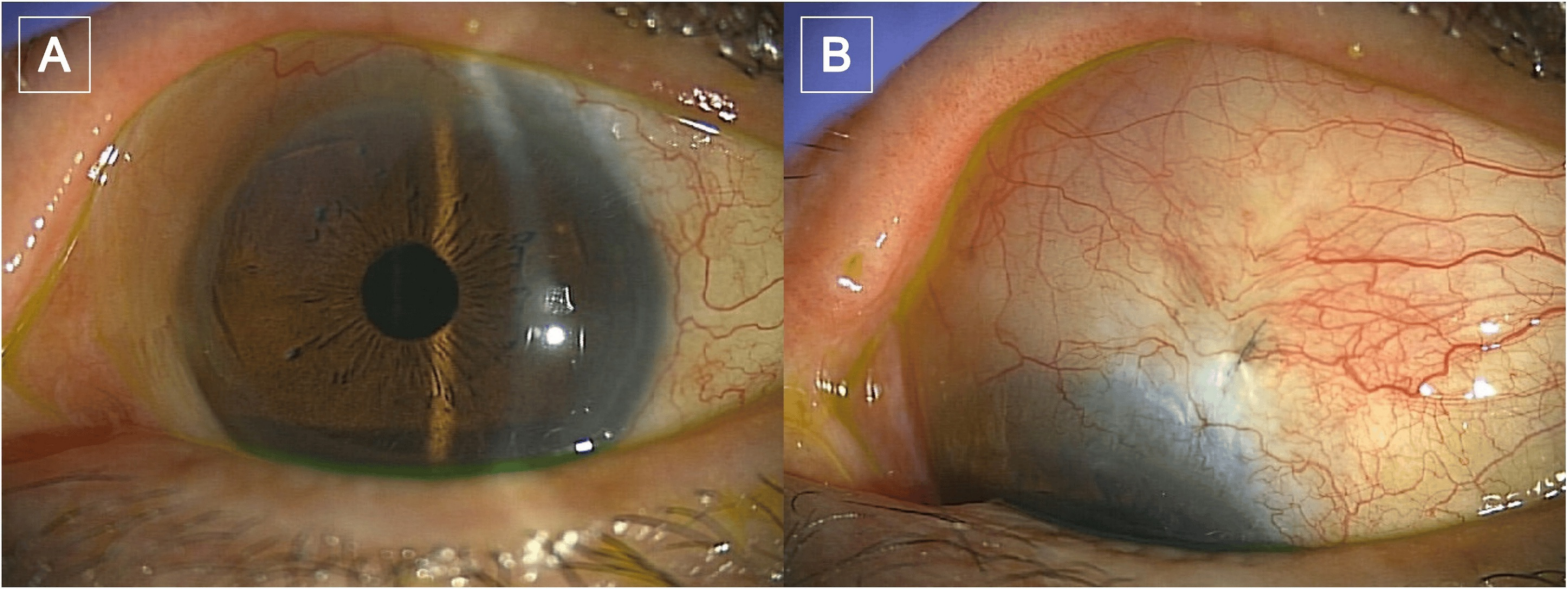
Post-revision outcome Two months after bleb revision: (A) Slit-lamp photograph showing a stable anterior segment without conjunctival injection. (B) Slit-lamp photograph showing complete conjunctival epithelialization with very well-healed tissue coverage

Two months after bleb revision, the conjunctiva was completely healed (Figure 2), BCVA was 1.0, and IOP was 16 mmHg with the use of a latanoprost-timolol fixed-combination eye drop and without any residual sequelae.

The detailed postoperative course is summarized in Table 1.

**Table 1:** Timeline of clinical course, interventions, specimen collection, and microbiology after PRESERFLO™ MicroShunt implantation POD: postoperative day; AC: anterior chamber; IV: intravenous; MRSA: methicillin-resistant Staphylococcus aureus; PMS: PRESERFLO™ MicroShunt; IOP: intraocular pressure; q2h: every 2 hours; q12h: every 12 hours; qHS: once nightly (at bedtime)

POD	Clinical findings	Interventions and treatments	Specimen collection	Microbiology/results
6		Intraluminal stent removed	–	–
18	Seidel test positivity	Conjunctival sutures removed; Ofloxacin ophthalmic ointment qHS added	–	–
25	Conjunctival hyperemia; mucopurulent discharge; eyelid swelling; whitish bleb opacification and swelling of the bleb; Seidel test positivity; no AC inflammation	Topical moxifloxacin + cefmenoxime q2h; ofloxacin ophthalmic ointment qHS; topical betamethasone discontinued	Conjunctival discharge	Direct smear: Gram-positive cocci
27	Expanded purulent infiltration; mild AC cells	Intracameral vancomycin (1 mg/0.1 mL) + ceftazidime (2.25 mg/0.1 mL)	Aqueous humor	
28	Partial improvement of ocular inflammation and eyelid swelling	Vancomycin ophthalmic ointment 1% 6×/day; IV vancomycin 1 g q12h ×4 doses + one dose after 24 h; levofloxacin eye drops 6×/day	–	Conjunctival discharge culture: MRSA (final)
32	Tube exposure	PMS removal; scleral tunnel sutured; conjunctival advancement; postop levofloxacin drops, vancomycin ointment, topical betamethasone (tapered)	–	Aqueous humor culture: no growth (final)
2 months after bleb revision	Quiet anterior segment; complete conjunctival epithelialization	latanoprost–timolol fixed combination for IOP control	–	–

## Discussion

There are very few reports of BRI following PMS implantation in the literature; i.e., only two cases have been described to date. Of those two, one involved Staphylococcus capitis, occurring three months after PMS implantation and presenting with a positive Seidel test result [11], and the other was caused by Staphylococcus epidermidis, which developed about four days after needling revisions [12]. In our case, blebitis developed at three weeks postoperatively and was accompanied by a positive Seidel test finding, thus suggesting that bleb leakage rather than intraoperative inoculation was the likely source of infection. Notably, this infection occurred despite routine postoperative prophylaxis with topical antibiotics and was caused by MRSA. To the best of our knowledge, this is the first documented case of MRSA blebitis in the early postoperative period following PMS implantation.

Compared with traditional trabeculectomy, PMS implantation, which is classified as a minimally invasive bleb surgery (MIBS) [14], is thought to confer a lower risk of BRI due to several key design and morphological differences. First, PMS implantation requires no scleral flap or sutures and uses a minimally invasive ab externo approach [8], which results in less disruption of conjunctival integrity and a more stable posteriorly-located bleb formation. Second, PMS blebs tend to have thicker walls, fewer conjunctival cysts, and reduced cavernous changes compared to trabeculectomy blebs [15], providing a more robust barrier against microbial ingress. And third, previous reports indicate that PMS implantation is associated with significantly fewer bleb-related complications and reinterventions than trabeculectomy [9,10], which reflects its gentler postoperative course and lower susceptibility to infections.

Although the unique morphological features of PMS blebs are thought to reduce the likelihood of late-onset BRI, early-onset BRI remains a concern, primarily due to bleb leakage. Prolonged topical glaucoma therapy can induce chronic structural and inflammatory changes in the conjunctiva and the Tenon’s capsule [16,17], thereby weakening their integrity and barrier function. In addition, PMS implantation universally requires intraoperative MMC to suppress subconjunctival fibrosis and ensure bleb function. However, excessive antifibrotic modulation can impair normal reparative processes, resulting in attenuated epithelial coverage, loss of fibrovascular support, and replacement of collagen with a loosely organized myxoid stroma [18]. 

Such alterations compromise tissue integrity and may predispose to an early bleb leakage. These two factors, chronic ocular surface compromise from prior medications and intraoperative antifibrotic modulation, can synergistically create structurally fragile blebs that are vulnerable to early leakage, despite the inherent design advantages of PMS that reduce late-onset complications. Although MMC 0.4mg/mL is routinely applied for five minutes in our practice [13], in this case, the duration may have been too long.

It should be noted that there is no standardized management protocol for BRI after MIBS. In managing this present case, we referred to the established treatment protocol for BRI following trabeculectomy [19]. Initially, the clinical findings were consistent with stage 1 blebitis, and the frequency of topical antibiotic instillation was increased, and bedtime application of antibiotic ointment was continued. As anterior chamber inflammation subsequently developed, the condition was considered to have progressed to stage 2, prompting intracameral antibiotic injection. Following culture confirmation of MRSA, both systemic and topical vancomycin were administered, leading to gradual clinical improvement. In Japan, topical vancomycin is commercially available in the form of an ophthalmic ointment, the clinical utility of which we have previously reported [20].

The infection appeared to be controlled without surgical intervention; however, the device needed to be removed due to the tube becoming exposed. In a previous study, explantation of the device was also performed, and bacteria were reportedly isolated directly from the explanted device [11]. In our case, no culture of the implant was performed, and it remains uncertain as to whether or not the device itself harbored the pathogen. Nevertheless, our experience suggests that in cases of BRI following PFM implantation, eventual tube removal may be unavoidable. Although the ocular findings showed gradual improvement with intensive antibiotic therapy, careful monitoring was continued until the infection was fully controlled.

## Conclusions

The findings in this report highlight that BRI can occur following PMS implantation, a procedure generally regarded as carrying a lower risk of infection compared with traditional trabeculectomy. Despite the distinctive bleb morphology and surgical advantages of PMS, early postoperative infection remains a potential risk, particularly in the presence of bleb leakage. Hence, clinicians should be mindful that blebitis can develop even when standard postoperative topical antibiotics are administered. In such cases, the potential involvement of resistant organisms, including MRSA, should be considered to ensure timely and effective management.

## References

[1] Razeghinejad MR, Havens SJ, Katz LJ (2017). Trabeculectomy bleb-associated infections. Surv Ophthalmol.

[2] Yassin SA (2016). Bleb-related infection revisited: a literature review. Acta Ophthalmol.

[3] Yamamoto T, Kuwayama Y, Kano K, Sawada A, Shoji N (2013). Clinical features of bleb-related infection: a 5-year survey in Japan. Acta Ophthalmol.

[4] Kangas TA, Greenfield DS, Flynn HW, Jr. Jr., Parrish RK, 2nd 2nd, Palmberg P (1997). Delayed-onset endophthalmitis associated with conjunctival filtering blebs. Ophthalmology.

[5] Jampel HD, Quigley HA, Kerrigan-Baumrind LA, Melia BM, Friedman D, Barron Y (2001). Risk factors for late-onset infection following glaucoma filtration surgery. Arch Ophthalmol.

[6] Rai PA, Barton K, Murdoch IE (2017). Risk factors for bleb-related infection following trabeculectomy surgery: ocular surface findings-a case-control study. Br J Ophthalmol.

[7] Ramakrishnan R, Bharathi MJ, Maheshwari D, Mohideen PM, Khurana M, Shivakumar C (2011). Etiology and epidemiological analysis of glaucoma-filtering bleb infections in a tertiary eye care hospital in South India. Indian J Ophthalmol.

[8] Ahmed II, Sadruddin O, Panarelli JF (2023). Subconjunctival filtration in evolution: current evidence on MicroShunt implantation for treating patients with glaucoma. Eye Vis (Lond).

[9] Pillunat KR, Herber R, Haase MA, Jamke M, Jasper CS, Pillunat LE (2022). PRESERFLO™ MicroShunt versus trabeculectomy: first results on efficacy and safety. Acta Ophthalmol.

[10] Khan A, Khan AU (2024). Comparing the safety and efficacy of Preserflo Microshunt implantation and trabeculectomy for glaucoma: a systematic review and meta-analysis. Acta Ophthalmol.

[11] Kudsieh B, Almazan-Alonso E, Ruiz-Medrano J, Samaan M, Ruiz-Moreno JM (2025). Staphylococcus capitus blebitis following PRESERFLO® MicroShunt implantation. J Glaucoma.

[12] Brambati M, Bettin P, Ramoni A, Battista M, Bandello F (2022). A case of endophthalmitis following needling procedure after PRESERFLO(®) Micro Shunt implantation. Eur J Ophthalmol.

[13] Mieno H, Mori K, Yoshii K, Okada Y, Ikeda Y, Ueno M, Sotozono C (2025). Risk factors and protective strategies for hypotony following preserflo microshunt implantation. Sci Rep.

[14] (2024). Minimally invasive bleb surgery for glaucoma: a health technology assessment. Ont Health Technol Assess Ser.

[15] Hasan SM, Theilig T, Meller D (2023). Comparison of bleb morphology following PRESERFLO(®) MicroShunt and trabeculectomy using anterior segment OCT. Diagnostics (Basel).

[16] Gozawa M, Takamura Y, Iwasaki K, Arimura S, Inatani M (2020). Conjunctival structure of glaucomatous eyes treated with anti-glaucoma eye drops: a cross-sectional study using anterior segment optical coherence tomography. BMC Ophthalmol.

[17] Lee S, Park DY, Huh MG, Cha SC (2024). Influence of preoperative glaucoma medication on long-term outcomes of trabeculectomy. Sci Rep.

[18] Elner VM, Newman-Casey PA, Patil AJ (2009). Aberrant wound-healing response in mitomycin C-treated leaking blebs: a histopathologic study. Arch Ophthalmol.

[19] Shoji N, Arakaki Y, Nakamoto K, Yamamoto T, Kuwayama Y (2018). Efficacy of predetermined therapeutic measures against bleb-related infection in the Collaborative Bleb-related Infection Incidence and Treatment Study. Acta Ophthalmol.

[20] Sotozono C, Fukuda M, Ohishi M (2013). Vancomycin Ophthalmic Ointment 1% for methicillin-resistant Staphylococcus aureus or methicillin-resistant Staphylococcus epidermidis infections: a case series. BMJ Open.

